# Retrotransposons Manipulating Mammalian Skeletal Development in Chondrocytes

**DOI:** 10.3390/ijms21051564

**Published:** 2020-02-25

**Authors:** Satoshi Kubota, Takanori Ishikawa, Kazumi Kawata, Takako Hattori, Takashi Nishida

**Affiliations:** Department of Biochemistry and Molecular Dentistry, Okayama University Graduate School of Medicine, Dentistry and Pharmaceutical Sciences, Okayama 700-8525, Japan; p1096qhn@s.okayama-u.ac.jp (T.I.); ph092dix@okayama-u.ac.jp (K.K.); hattorit@md.okayama-u.ac.jp (T.H.); tnishida@md.okayama-u.ac.jp (T.N.)

**Keywords:** retrotransposon, endogenous retrovirus, chondrocyte, cartilage, skeletal development

## Abstract

Retrotransposons are genetic elements that copy and paste themselves in the host genome through transcription, reverse-transcription, and integration processes. Along with their proliferation in the genome, retrotransposons inevitably modify host genes around the integration sites, and occasionally create novel genes. Even now, a number of retrotransposons are still actively editing our genomes. As such, their profound role in the evolution of mammalian genomes is obvious; thus, their contribution to mammalian skeletal evolution and development is also unquestionable. In mammals, most of the skeletal parts are formed and grown through a process entitled endochondral ossification, in which chondrocytes play central roles. In this review, current knowledge on the evolutional, physiological, and pathological roles of retrotransposons in mammalian chondrocyte differentiation and cartilage development is summarized. The possible biological impact of these mobile genetic elements in the future is also discussed.

## 1. Introduction

Supported by remarkable advances in biotechnology and bioinformatics, the primary digital codes recorded in the genomes in creatures on Earth have been rapidly deciphered. As a result, a number of novel features of mammalian genomes were uncovered, most of which were against the expectation of scientists. For example, the number of protein-coding genes in the human genome turned out to be approximately 20,000 [1], which is comparable to, and even less than, that in the murine genome. Regarding the organization of the human genome, the area used for encoding those proteins is as much as 2%, whereas 55% of the whole genome is occupied by repetitive sequences [2]. Considering that primordial eukaryotic genomes are much smaller and contain much fewer repetitive elements, it is clear that the expansion of repetitive elements has played a critical role in the development of the human genome during the course of mammalian evolution.

**Figure 1:**
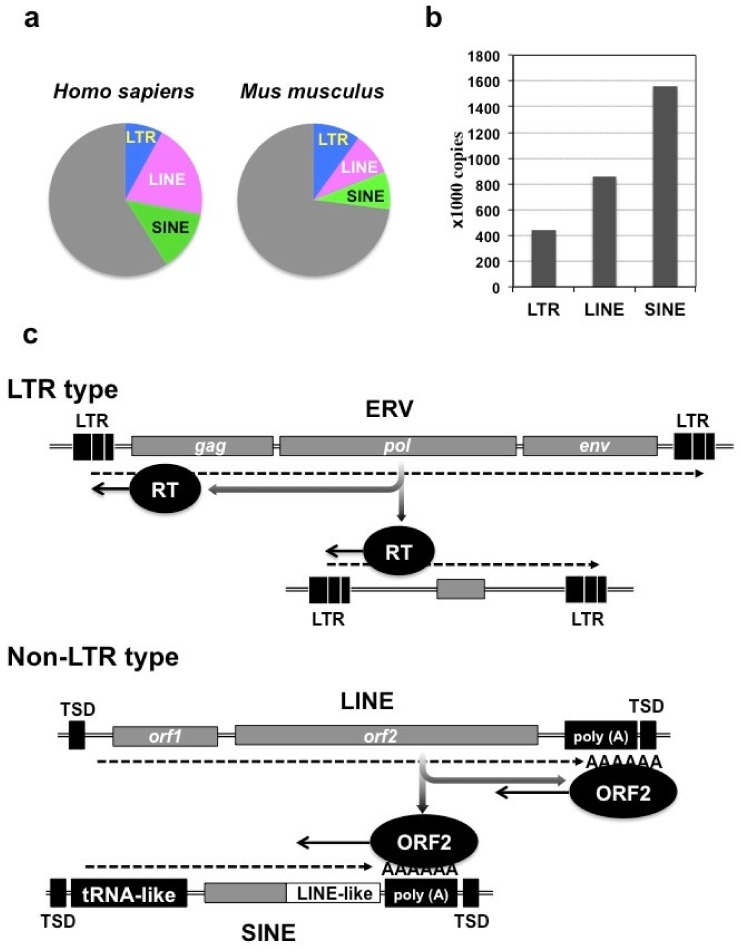
Retrotransposons in the human genomes. (**a**) Pie graphs showing the occupancy of retrotransposons in the human and murine genomes. (**b**) Copy numbers of three types of retrotransposons in the human genome. (**c**) Structure and mode of transcription (dotted lines with arrows) and reverse transcription (solid lines with arrows) of each type of retrotransposon. LTR, long terminal repeat; ERV, endogenous retrovirus; LINE, long interspersed nuclear element; SINE, short interspersed nuclear element; RT, reverse transcriptase; ORF2, open reading frame protein 2 of LINE; TSD, target site duplication. For ERV, a complete provirus retaining all of the essential retroviral genes, *gag, pol,* and *env*, is shown as a representative.

Expansion of repetitive sequences can be achieved at the level of DNA replication. However, the contribution of such a process at a DNA level to human evolution seems to be limited, since most of the repetitive sequences in the human genome originate from mobile genetic elements entitled retrotransposons multiplying through the reverse-transcription process (Figure 1a) [2].

Retrotransposons are endogenous genetic elements that produce their copies and insert them randomly in the host genome. Following regular transcription to RNA, their DNA copies are produced by reverse transcription using the RNA as a template. This “DNA–RNA–DNA” replication cycle may not appear sophisticated, but it is a fundamental process that formed the present DNA world. Given that the original self-replicating genetic material on Earth was RNA [3,4], any RNA gene that has created the DNA world must have been reverse-transcribed to form a corresponding DNA copy, which was followed by RNA transcription. Naturally, the nascent RNA copy could be copied again to form another DNA copy. During the transitional era from the RNA world to the DNA world, the DNA–RNA–DNA replication cycle must have been the central process to form and enlarge the DNA genome. Following this classical genome manufacturing strategy, retrotransposons have constructed our genome over several hundred million years [5].

Reverse transcriptase was first discovered in the Rous sarcoma virus (RSV), an avian oncogenic retrovirus, in 1972 [6]. At that time, these retroviral enzymes were regarded as living fossils, and thus we failed to discover our reverse transcriptases produced from our own genomes for a long time. However, we now know that retrotransposons are still manipulating our genome, which may lead to further animal evolution, using our own reverse transcriptases. In spite of its biological significance, the roles of these internal gene-editing units in chondrogenesis and skeletal development are not widely recognized. Therefore, in this review article, current knowledge on the biological impact of these mobile genetic elements in chondrocytes of present mammals, which occasionally affects the structure and function of skeletal elements, is summarized and discussed, after a brief introduction of the retrotransposons still active in our genomes. 

## 2. Retrotransposons in the Mammalian Genome

The mammalian genome contains two major classes of mobile genetic elements, and retrotransposons fall into the class 1 category [2]. Class 2 mobile genetic elements represent DNA transposons, which travel in the host genome by cutting and pasting themselves. Although the human genome contains 294,000 copies of DNA transposon-associated elements, they are no longer active and are regarded as genetic fossils [2]. In contrast, class 1 mobile genetic elements include a number of active retrotransposons that are able to modify our genome structure. These retrotransposons are classified into two major groups; one is long terminal repeat (LTR) type, and the other is non-LTR type [2,5]. Both groups contain autonomous retrotransposons that can copy and paste themselves using their own devices, and non-autonomous retrotransposons that can replicate only under the assistance of the autonomous ones. The autonomous non-LTR retrotransposons are represented by long interspersed nuclear elements (LINEs), whereas the non-autonomous ones are called short interspersed elements (SINEs). Among mammalian species, the human genome is particularly characterized by the involvement of a vast number of retrotransposons (Figure 1a). Indeed, more than 40% of the human genome is occupied by these mobile genetic elements, in which the LINE territory is the largest. In contrast, their occupancy in the murine genome is approximately 27%. This discrepancy suggests a critical role of these elements during the course of human evolution [2]. Regarding the copy number in the human genome, SINE stands out, represented by the *Alu* family that was actually regarded as a hallmark of the human genome (Figure 1b) [7].

The LTR-type retrotransposons are characterized by the two LTRs at both ends (Figure 1c). These elements evolved from a retroviral provirus integrated into the host genome at a certain time point during evolution, and thus are generally entitled endogenous retroviruses (ERVs). A prototypic ERV proviral genome retains *gag, pol*, and *env* genes, which are necessary for nucleocapsid formation, reverse transcription/integration, and infection/transmission, respectively. After transcription, genomic RNA is reverse transcribed from the primer binding site located immediately downstream of the LTR at the upstream end, using a host tRNA as a primer. Complete cDNA is synthesized from the genomic RNA through two rounds of jumping via the “R” region in the LTRs [8]. Reverse transcriptase produced by *pol* gene is also utilized for the propagation of non-autonomous LTR-type elements lacking protein-coding genes. Since retrotransposition is possible even without *env*, a significant number or ERV proviruses do not retain *env* at present [2]. The human genome holds a large family of human ERVs (HERVs) with known and unknown functions. Of note, expression of a number of HERV subfamily H (HERV-H) members was found induced transiently upon the establishment of induced pluripotent stem (iPS) cells from somatic cells [9]. Furthermore, a recent study indicated that these active HERV-H retrotransposons create topologically associating domains (TADs) in pluripotent stem cells [10]. Interestingly, mice also possess LTR-type retrotransposon fossils entitled mouse endogenous retrovirus type-L (MuERV-L) elements that are actively transcribed at early stages of embryonic development [11]. Together, these findings indicate the functional contribution of ERVs in mammalian development, as well as evolution.

The non-LTR autosomal LINEs contain two protein-coding regions, in which an internal ribosomal entry signal (IRES) is utilized for the translation of the second open reading frame (*orf2*). The *orf2* encodes a protein (ORF2) with reverse transcriptase activity. Polyadenylated LINE transcripts are reverse transcribed by ORF2 from the poly (A) tail, while ORF2 also recognizes an AT-rich tract in the host genome and cuts it. As such, reverse transcription and integration of LINESs are both conducted by ORF2 protein (Figure 1c). Upon integration, duplication of the short nucleotide sequence at the integration site occurs, leaving target site duplication (TSD) at both ends [12]. SINEs probably share the same origin with tRNAs, since these elements retain the structural characteristics of tRNA and are transcribed by RNA polymerase III. These transcripts do not contain any open reading frames, but involve LINE-like polyadenylated regions to mimic LINE transcripts. Utilizing this region, SINE transcripts are able to summon ORF2 produced by LINE to exploit this protein for self-expansion [13]. The human genome is filled with nearly one million *Alu* elements, which are 7SL RNA-derived SINEs. Moreover, *Alu* elements are still increasing their de novo copies in our genome, for example, in the locus of breast cancer susceptibility gene 1 (BRCA1) [14]. This gene that encodes a tumor suppressor is already suffering from insult by a tremendous number of *Alu* elements. Similarly, the murine genome contains over a million SINE elements represented by 7SL RNA-derived SINE B1 and tRNA-derived SINE B2. Advances in molecular genomics would reveal more species-specific distribution of retrotransposons in the genomes of other mammalian species. 

## 3. Creation and Modification of Genes by Retrotransposons

### 3.1. Modification of Pre-existing Host Genes by Retrotransposons

Retrotransposons, particularly LTR-type ones, insert their DNA copies anywhere in the host genome without preference in nucleotide sequences. However, the integration site may be delimited epigenetically by chromatin status. Namely, actively transcribed loci in euchromatin are more accessible for these mobile elements than those condensed in heterochromatin. Thus, unfortunately, active genes with significant biological functions are exposed to the risk of genetic manipulation by retrotransposons. 

**Figure 2:**
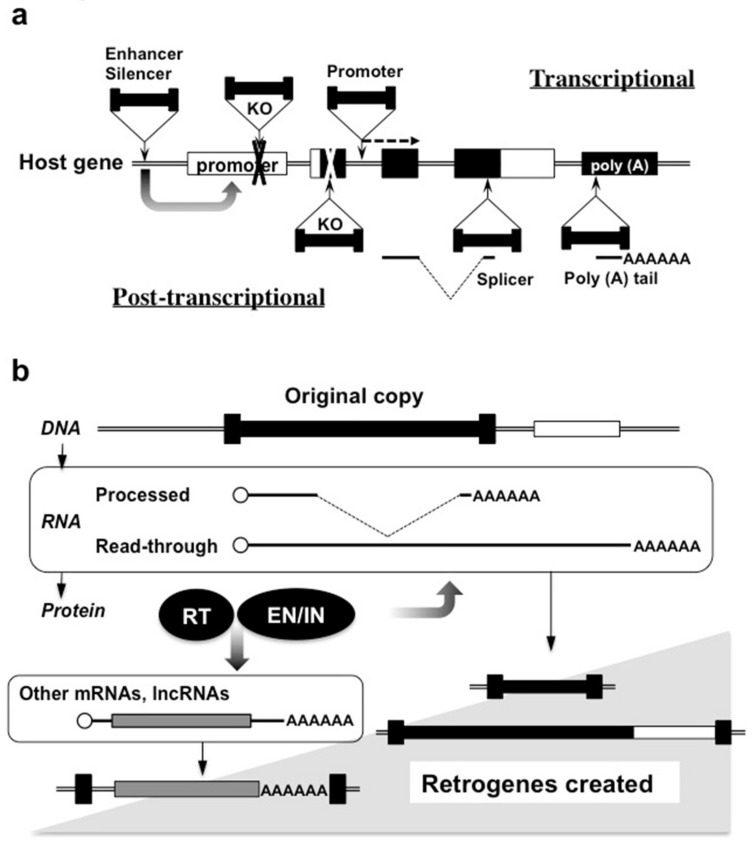
Modification and creation of genes by retrotransposons. (**a**) Modification of pre-exiting host genes by the integration of transposable genetic elements. Transcriptional and post-transcriptional effects are summarized above and below the target host gene, respectively. Additional genetic elements provided by retrotransposons are noted, occasionally causing destruction of the target gene (KO). (**b**) Creation of novel genes by retrotransposons. RT and EN/IN represent proteins with reverse transcriptase and endonuclease/integrase activities, which are encoded by retrotransposon mRNAs, respectively.

The insertion of mobile elements confers remarkable impact on the behavior and product of target genes at both transcriptional and post-transcriptional levels (Figure 2a) [15]. Destruction of the authentic promoter by such genetic intruders results in loss of function of the target gene at the transcriptional level. Conversely, insertion of enhancers and promoters, which are typically built in LTR, occasionally and differentially adds extra regulatory machinery to the target gene, depending upon the integration site. If a retrotransposon-derived promoter is added downstream of the open reading frame, novel proteins can be produced. This also happens when the mobile elements are inserted, destroying the initiation codon, causing a frame-shift of the reading frame, or inducing alternative splicing by the addition of extra splicing junctions. The stability of the host mRNA can also be modified by the addition of a retrotransposon-derived alternative poly (A) tail. As such, the genetic impact of retrotransposition is much more complex and can be more serious than DNA damage and subsequent mutation caused by errors and environmental carcinogens. It should be noted that naked moles, which only have fossil transposons and are lacking active ones at present, are characterized by extraordinary longevity without developing cancers [16].

### 3.2. Creation of Novel Genes by Retrotransposons

Along with the expansion in the host genome, retrotransposons accumulate mutations and gradually lose their proper gene products. During this process itself, new protein coding genes and, more likely, new long noncoding RNA (lncRNA) genes are occasionally created. In particular, the contribution of retrotransposon to lncRNA gene formation is prominent—for 75% of human lncRNA contains retrotransposon-derived sequences, at least in part [2]. These data are consistent with the fact that the human genome contains over 27,000 lncRNA genes, which is much more than that in the murine genome with less retrotransposon occupancy [17]. In addition to the genes directly evolved from retrotransposons, formation of chimeric genes containing both retroviral and host fragments is widely recognized. A classic example can be found in viral oncogenes created by animal retroviruses. RSV contains an oncogene known as *v-src* under the control of a strong viral promoter in the LTR, which transforms infected cells into sarcoma. The *v-src* gene is homologous to the host *c-src* gene and located downstream of the retroviral *gag–pol–env* cistron related to avian leukosis virus (ALV). This genomic organization suggests that RSV genomic RNA originated from a chimeric product of the parental ALV provirus with the cellular *c-src* gene located immediately downstream [18]. Finally, both retrotransposon-derived and host-derived portions of transcripts can create processed genes through the reverse transcription and integration of splicing variants of the original transcripts, adding more diversity to the host genome organization.

Apart from the genes constructed in cis by retrotransposons, which were introduced above, LINEs can manufacture novel genes with host mRNAs in trans, leaving only a slight trace of their work in the new genes. These resultant genes usually contain a processed cDNA of a host mRNA, and thus are called retrogenes. As described in Figure 1c, the ORF2 protein of LINEs reverse transcribes their RNAs recognizing their poly (A) tails. Therefore, theoretically, ORF2 could produce a cDNA from any poly (A)-containing host mRNA (Figure 2b). Nevertheless, this does not happen frequently for ORF2 strongly prefers its own mRNA (= LINE genomic RNA) as the template for reverse transcription [2]. Without this cis-preference, LINEs should have been serious threats to mammalian genome maintenance.

## 4. Gene Modification by a LINE Leading to Chondrodysplasia in Mice

In 2004, a mouse mutant with hereditable skeletal dysplasia and male infertility was first reported [19]. This mouse, named *chagun*, arose spontaneously and harbored the locus responsible for this phenotype in chromosome 9, which corresponds to human chromosome 3. In this mouse, disproportionate growth is observed in the skull and other skeletal parts. For example, the femora of *chagun* mice are significantly smaller, with disorganized growth plates. At birth, the mutant growth plate is apparently normal; however, disorganization in the growth plate progresses along with age. Abnormality in the growth plate is prominent in the proliferative zone. The columnar structure of flat proliferating chondrocytes becomes deranged with the morphological alteration in each chondrocyte. The intracellular position of Golgi apparatus in *chagun* chondrocytes suggests that cellular polarity is perturbed. Finally, a significant number of these chondrocytes undergo apoptosis, leading to growth insufficiency of long bones.

Subsequent genetic investigation identified the gene responsible for this *chagun* phenotype [20]—the *Poc1a* encoding protein of centriole 1A. As the name implies, this protein is a component of centrosome, suggesting its potential function in microtubule organization. Consistent with this anticipation, defects in the primary cilium and the mitotic spindle were evident in this mutant mouse, which led to craniofacial and skeletal abnormalities through impaired polarity of growth plate chondrocytes.

**Figure 3:**
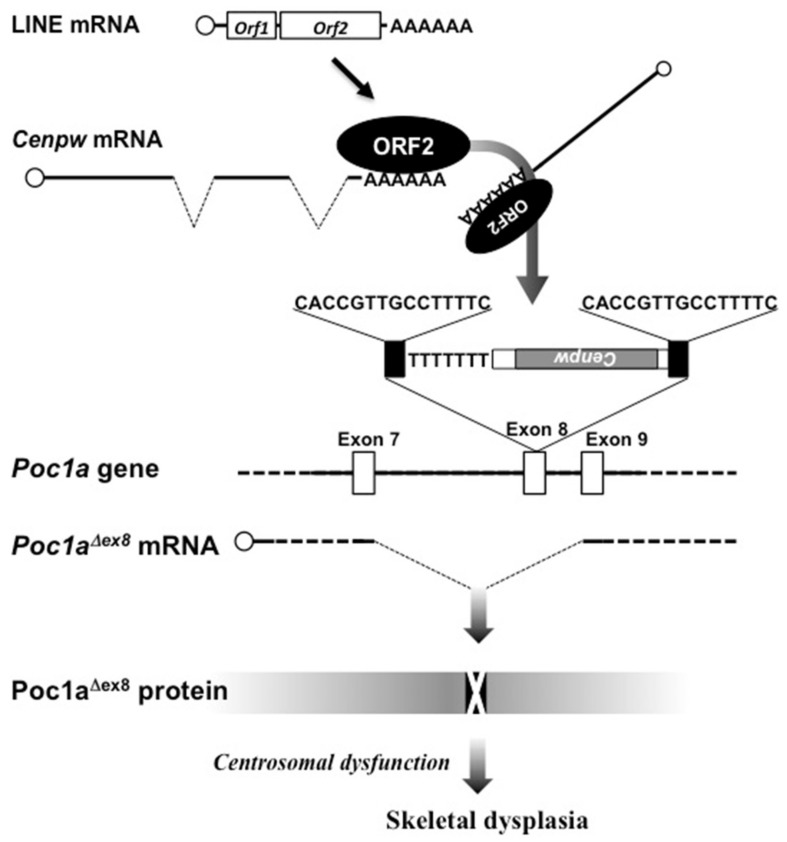
Exon skipping in *Poc1a* caused by LINE-1 in *chagun* mice. The nucleotide sequences of the target site duplications are shown in the middle. LINE, long interspersed nuclear element; ORF2, open reading frame protein 2 of LINE; *Cenpw*, centromere protein W gene; *Poc1a*, protein of centriole 1a gene. Polyadenylated *Cenpw* processed gene insertion occurred in a direction reverse to that of *Poc1a*, forming an apparently poly (T) nucleotide sequence.

Of note, molecular genetic analysis revealed that *Poc1a* was destroyed by the insertion of a cDNA element in the middle of exon 8, which caused the skipping of this exon upon mRNA splicing to produce POCA1 protein lacking biological activity (Figure 3). The intruded DNA element was not of a LINE itself, but was a processed gene of centromere protein W (*Cenpw*) from chromosome 10. Nevertheless, clear traces of LINE-1 (a major group of LINEs)-mediated retrogene transposition were left. The first one was the poly (A) tail located downstream of the *Cenpw* cDNA, and second one was a set of TSDs at both ends of the integrated element. These findings indicate that ORF2 protein reverse transcribed and inserted the *Cenpw* cDNA into the *Poc1a* locus in *chagun* mice.

Several human pedigrees harbor mutations in *POC1A* loci, commonly characterized by growth insufficiency [20,21]. Therefore, the *chagun* mice phenocopying human cases can be a good model for medical investigation of these relevant human syndromes. This mouse also provides precious information about the role of the primary cilia in chondrocytes and skeletal development. Another interesting point is that *chagun* mutation affects the function of Sertoli cells, as well as that of chondrocytes. Although these two types of cells exert totally different missions in spermatogenesis and skeletal development, these are both SOX9-positive. Investigation on this SOX9–POCA1 axis may reveal novel aspects of the mechanism of skeletal growth and reproduction.

## 5. Creation of Short-legged Dogs: New *FGF4* Gene Formation by LINE-1

Surprisingly, LINEs helped the evolution of dogs as partners of human beings. Dogs and cats are the two major partner animals for us, and particularly in city areas, small and short-legged breeds are often preferred, since they are relatively easy to control and require less space. Pembroke Welsh corgis, basset hounds, and dachshunds are widely known as typical examples of such dogs; however, it may not be recognized well that LINE-1 is responsible for at least 19 breeds of them.

**Figure 4:**
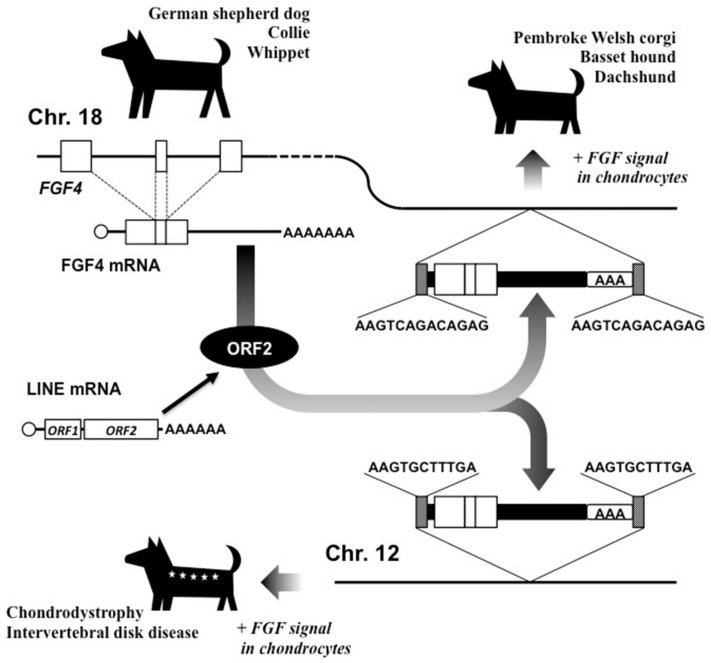
Creation of two new *FGF4* retrogenes in distinct genomic context causing short legs and intervertebral disc diseases in dogs. Proper target site duplication sequences (stippled boxes) are shown for each integration site. Open and solid boxes denote FGF-4 encoding regions and untranslated regions, respectively, together with poly (A) tail (AAA).

Unlike the case of *chagun* mice, these short-legged dogs were created not by the destruction of a certain gene by LINE-associated intrusion, but by the addition of a processed host gene that encoded a functional growth factor [22]. In their genomes, an extra fibroblast growth factor (FGF) 4 cDNA containing all of the exons, including full coding regions and the 3’-UTR, was inserted in chromosome 18, in which the original *FGF4* gene was present (Figure 4). The cDNA was followed by a poly (A) tail and was flanked by TSDs at both ends, clearly indicating the contribution of LINE-1 to its integration. This cDNA retains exactly the same protein-coding nucleotide sequence as that of the parental *FGF4* gene, suggesting that it is required and conserved for the short-legged phenotype. This evolutional event caused by LINE-1 was confirmed, not only in the four breeds introduced above, but also in another 15 breeds in domestic dogs. In fact, FGF signaling is known to be closely associated with skeletal development. In humans, activating mutations in FGF receptor 3 gene (*FGFR3*), which is the major receptor transmitting FGF signals in chondrocytes, cause achondroplasia with typical dwarfism phenotype [22,23]. Therefore, LINE-1 may have done, or may do in the future, the same thing in humans, causing skeletal growth insufficiency. However, fortunately in this case, LINE-1 has established these major groups of domestic dogs that are widely accepted as excellent human partners through a single evolutionary event by adding an extra FGF signal emitter.

Another intriguing report suggests that breeds with additional *FGF4* retrogenes were not necessarily established under a strong selective human pressure for preference of short-legged dogs. A genome-wide association study of skeletal dysfunction and intervertebral disc disease in Nova Scotia duck tolling retrievers (NSDTRs) uncovered a different *FGF4* retrogene in chromosome 12 as a causative element of these diseases (Figure 4) [24]. This *FGF4*-inserted genotype was significantly associated with the height and intervertebral disc disease (IVDD) in these dogs, and *FGF4* expression was nearly 220-fold higher in the neonatal intervertebral disc of dogs with homozygous *FGF4* retrogene insertion than those without, clearly indicating its etiological role.

Since the TSD sequences of these *FGF4* retrogenes in chromosomes 12 and 18 are distinct, these events were exerted independently by LINE-1 ORF2 capturing the same host mRNA, suggesting its remarkable activity in germ cells, or more possibly, in embryonic cells. Collectively, these two genetic events conducted by LINE-1 represent an awful property of a retrotransposon as a creator of endogenous pathogenic agents, using host mRNA as a template. Such an endogenous pathogen could even initiate hereditary diseases, as well as malignant neoplasms.

## 6. Hallmarks of Human Genome Regulating Chondrogenesis and Skeletal Development

### 6.1. Height-related Gene Evolved along with Alu, a SINE Highly Specific to Primates

Owing to its exclusive inclusion in the human genome, with its absence in the genomes of rodents and other mammalian species, *Alu* retrotransposon was believed to be a hallmark of the human genome. However, comparative molecular genomics revealed that the *Alu* SINEs are widely shared by other primate species, suggesting its contribution to the evolution of primates rather than that of *Homo sapiens*. Among a number of genes evolved with *Alu* elements, the zinc finger and broad complex/tram track/bric-a-brac 38 (ZBTB38) gene is known to be expressed in chondrocytes and involved in the determination of height in humans. This gene encodes a transcriptional repressor and is conserved among vertebrates, and its murine orthologue, C-terminal binding protein-interacting BTB zinc finger protein (CIBZ) gene, is shown to regulate apoptosis. A strong association between height and ZBTB38 locus was reported both in European and Korean populations [25,26], which may be ascribed to its regulatory function of apoptosis, possibly in growth plate chondrocytes.

**Figure 5:**
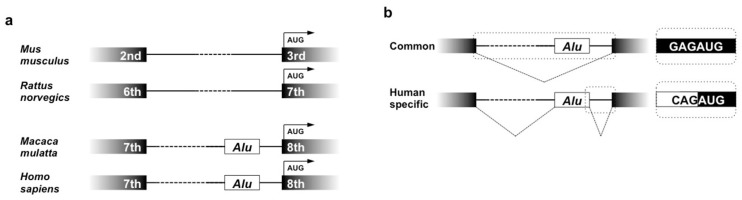
*Alu* insertion in primate ZBTB38 locus. (**a**) Structure of the rodent and the primate ZBTB38 gene and its orthologues around the translation initiation sites. Numbered exons and introns in between (lines) are illustrated with inserted *Alu* sequences (Open boxes). (**b**) Splicing patterns observed commonly among primates, and specifically in humans. The resultant variation in the nucleotide sequences around the translation initiation codon is shown on the right. The human-specific initiation codon displays a weaker Kozak’s consensus.

*Alu* integration into ZBTB38 occurred before the evolution of *Hominidae*, since it is already present at this particular locus in the genomes of the rhesus macaque (*Macaca malatta*) and the common marmoset (*Calithrix jacchus*) [27]. Although ZBT38 gene is conserved, the gene structure is different between primates and rodents (Figure 5a). The open reading frame starts from the 3rd and 7th exons of mouse and rat CIBZ genes, respectively, whereas primate ZBTB38 protein is encoded after the 8th exon. In the intron immediately upstream of the 8th one, an *Alu* copy is integrated. As such, the location of the *Alu* element in ZBTB38 is identical among primates. However, interestingly, these *Alu* elements behave differently, depending upon species. In the rhesus macaque and the common marmoset, splicing of ZBTB38 pre-mRNA is conducted as if the *Alu* element is not present, eliminating this element as a part of the intron. In contrast, in human cells, an alternative acceptor in the *Alu* element accepts the authentic donor of the 7th exon. The same *Alu* element also provides an alternative donor for the acceptor of the 8th exon. Consequently, the majority of the *Alu* sequence is added as an extra exon of 123 bases between exons 7 and 8 in the mRNA variant (Figure 5b) [27]. Since the *Alu*-derived new exon is located upstream of the translation initiation codon, it does not affect the primary structure of ZBTB38 protein. Instead, addition of this exon may alter the translation efficacy of mRNA by changing the proximity of the nucleotide sequence around the translation initiation codon to the Kozak’s consensus. It needs to be noted that the authentic mRNA with properly connected exons 7 and 8, as well as the *Alu*-containing one, is expressed in a variety of normal human cells. The impact of the *Alu* exonization on chondrocyte biology and skeletal development still remains unclear; however, the human-specific behavior of the *Alu* element implies a novel epigenetic mechanism of retrotransposon regulation in human cells and the human-specific role of such *Alu* elements.

### 6.2. Human Endogenous Retroviruses

As introduced in a previous subsection, the human genome is characterized by the involvement of a vast number of lncRNA genes that are even more abundant than the protein-coding classical genes. To date, although nearly 20,000 lncRNAs out of 27,000 in total are anticipated to play critical biological roles in the human body [17], the actual functionalities of most lncRNAs still remain to be clarified. HERVs produced a number of such lncRNA genes during their expansion along human evolution, which are transcribed under physiological and pathological status.

As is often the case with research regarding a novel class of molecules, an early advance in lncRNA research was also conducted in relation to cancer biology. Thus, a significant number of lncRNAs were found through cancer research and were defined as cancer-associated lncRNAs. Urothelial cancer-associated 1 (UCA1) is the most typical representative of such lncRNAs. Until today, UCA1 has been reported to be associated not only with bladder cancers [28,29,30], but also with other major human malignancies, including breast cancers [31,32,33]. The UCA1 gene is a typical LTR-type retrotransposon retaining *gag* and *pol* genes flanked by LTRs at both ends, lacking the *env* gene. This provirus is classified as part of the HERV-H subfamily and is located in the short arm of chromosome 19 in the human genome [34]. This HERV-H copy is hominid-specific, for orthologues can be found only in four species other than us—bonobos, chimpanzees, orangutans, and gorillas. Therefore, UCA1 is supposed to have contributed to human evolution and to have played a significant role in human-specific developmental events.

**Figure 6:**
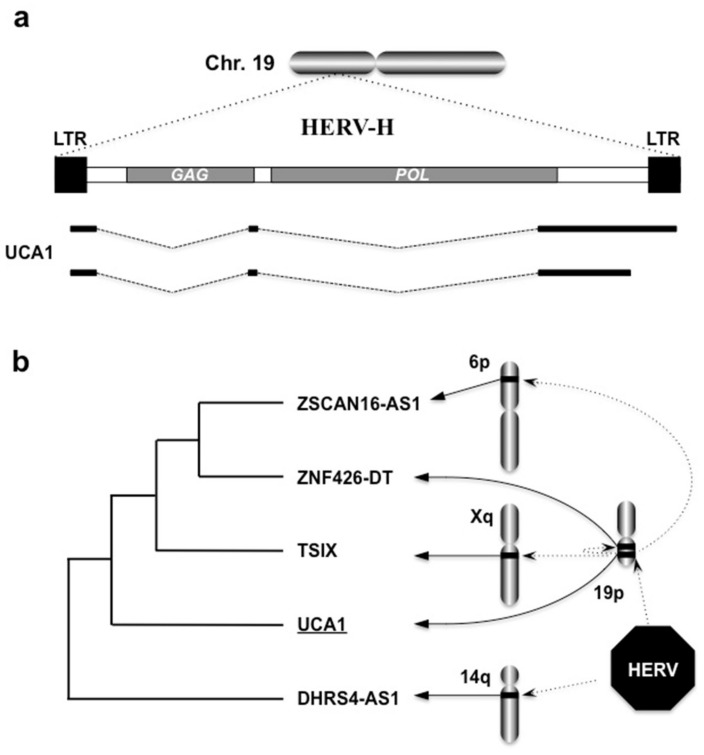
Generation of UCA1 by a human endogenous retrovirus subfamily H (HERV-H) provirus. (**a**) Structure and transcripts of the UCA1 gene in human chromosome 19. Between the two long terminal repeats (LTRs), two (*GAG* and *POL*) out of three retroviral genes are structurally retained. Two major transcript variants are illustrated. (**b**) UCA1-associated lncRNAs and their evolution. As a result of homology search with the basic local alignment search tool (BLAST), four homologues that are confirmed to be expressed and contain segments displaying ≥ 200 BLAST scores were identified in human Reference RNA Sequences. A phylogenetic tree was constructed by the fast minimum evolution method. All of them are lncRNAs and were probably processed and created from the same HERV ancestor (octagon). Chromosomal locations (solid arrows) and possible expanding routes (dotted arrows) of these UCA1-associated genes are illustrated as well.

In spite of the structural retention of *gag* and *pol* genes, the UCA1 gene expresses lncRNAs only. This gene is able to produce several lncRNA variants, which are represented by two major ones of approximately 1400 and 2300 bases in length (Figure 6a). One should note that the human genome possesses several active lncRNA genes that are closely related to the UCA1 gene. All of the lncRNA genes shown in Figure 6b are confirmed to be expressed in human cells [35,36,37,38]. These lncRNAs share a high degree of homology with UCA1, especially in the LTR regions. It is assumed that these genes originate from the same retroviral progenitor, and thus they can be regarded as members of a gene family. Since the UCA1 gene retains *gag* and *pol* genes, it may have copied and pasted itself to generate another family member on the way to humans at present (Figure 6b).

**Figure 7:**
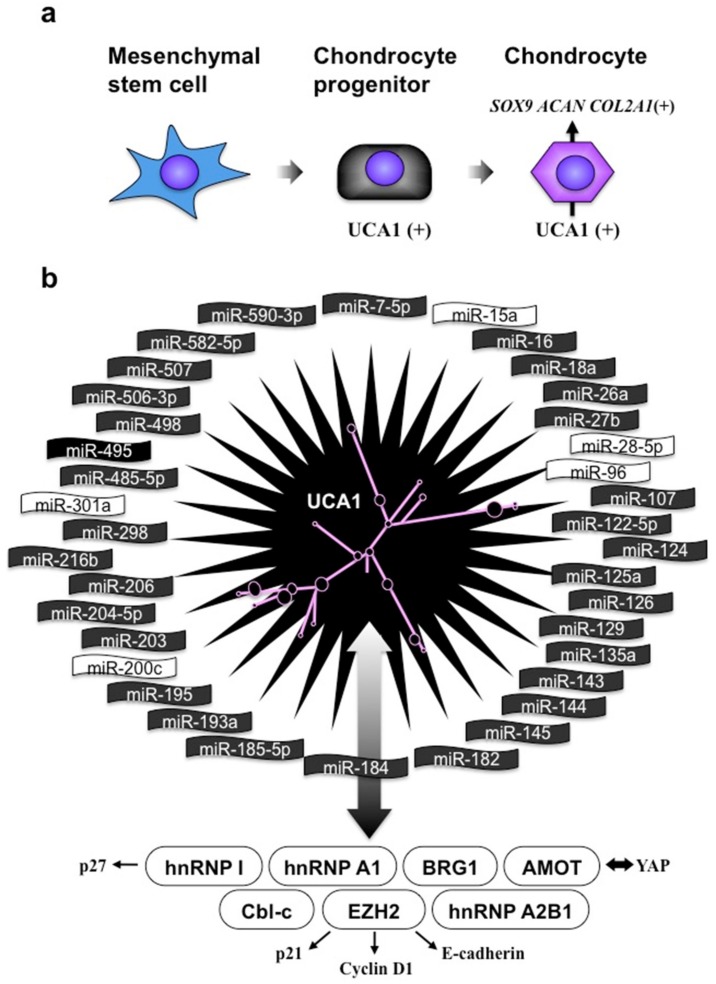
UCA1 functions. (**a**) Expression and function of UCA1 during chondrocytic differentiation. *SOX9*, *ACAN* and *COL2A1* are chondrocyte-specific genes. (**b**) Molecular counterparts supporting the multiple functions of UCA1. Around the predicted secondary structure of UCA1, miRNAs that are known to be under the regulation of UCA1 are clustered. The ones with their names in white were shown to directly bind to UCA1. At the bottom, proteins, which directly interact with UCA1, are summarized with their known targets. Regular arrows represent genetic targets of these proteins, whereas the bi-directional arrows indicate the direct molecular interactions.

UCA1 has been extensively investigated as a cancer-related lncRNA. However, recently, the physiological role of UCA1 in chondrocyte differentiation was uncovered [39,40]. During the process of differentiation from human bone marrow mesenchymal stem cells to chondrocytes, UCA1 was found to be induced rapidly and strongly prior to the induction of *SOX9*, a gene encoding the master transcription factor of chondrocytic differentiation. Furthermore, overexpression and silencing of the UCA1 gene resulted in the promotion and attenuation of chondrocyte differentiation, respectively, in human cells (Figure 7a). Interestingly, UCA1 could enhance the chondrogenesis, even in mouse chondroblasts that do not possess UCA1 genes. Considering the difference in skeletal sizes between hominids with UCA1 and rodents without UCA1, UCA1 may be acting as one of the determinants of skeletal size by regulating the endochondral ossification process.

Regarding the molecular function of UCA1, a vast number of recent reports have uncovered the functional complexity of this lncRNA (Figure 7b). As supposed by the predicted secondary structure (Figure 7b, center), UCA1 interacts with a variety of protein counterparts, using its secondary-structured functional elements as an interface. By interacting with these proteins, UCA1 occasionally acts as a scaffold for molecular events, sequesters them to prevent their works, or modifies their functions, conferring multiple biological outcomes [41,42,43,44,45,46,47,48]. Moreover, another function as a competitive endogenous RNA (ceRNA) may not be overlooked. Since UCA1 contains at least 32 experimentally verified binding sequences for distinct miRNAs, this single lncRNA can absorb these miRNAs away from the target mRNAs to de-repress the miRNA target genes [49,50,51,52,53,54,55,56,57,58,59,60,61,62,63,64,65,66,67,68,69,70,71,72,73,74,75,76,77,78,79,80,81,82,83,84,85]. Given that each miRNA in Figure 7b regulates thousands of target mRNAs, it is not easy to deduce the biological outcome caused by UCA1 via these miRNAs. However, it is strongly suggested that UCA1 should be playing significant roles in the process of endochondral ossification after chondrogenesis. Currently, it is at least clear that UCA1 is capable of promoting the maturation of proliferating chondrocytes via sponging miR-145 [86]. Indeed, HERV-H produced such a hominid-specific molecule with excessively multiple and context-dependent functions.

## 7. Retrotransposons in Human Joint Diseases

### 7.1. Osteoarthritis

Osteoarthritis (OA) is a multifactorial degenerative disease of the synovial joints and, currently, is a major medical issue in advanced countries with highly aged societies. Although OA itself is not life-threatening, progressive loss of locomotive function in patients severely impairs their quality of life. Unlike rheumatoid arthritis (RA; see the next subsection), OA is initiated from the loss of the integrity of articular cartilage in joints, most frequently in body weight-bearing knee and hip joints [87]. The fact that OA is usually developed in aged persons after a long time of mechanical loading on joints suggests continual mechanical stress as a critical etiological factor. Consistent with this notion, OA can quickly develop in young athletes, represented by soccer players, particularly after damage in the synovial ligaments, which allows traumatic movement of condyles. However, other associated etiological factors for OA development are being investigated as well, which include genetic background, systemic conditions, and viral infection.

In 2014, a Norwegian group attempted to determine if a virus was associated with OA, since a previous report [88] suggested human herpes virus 4 (HHV-4) infection might be specific to OA cartilage. As a result, they failed to detect any transcript from parvovirus B19, hepatitis C virus, or human herpes viruses in cartilage and cultured chondrocytes from OA patients. Nevertheless, they were able to detect several transcripts from HERV-W therein instead [89]. One of the transcripts corresponding to the *env* mRNA of HERV-WE2 was found expressed in 88% of advanced OA chondrocytes, while it was absent in normal and early OA chondrocytes. The etiological role of the activation of this particular subgroup of HERV is not yet clear. Since HERV expression is occasionally induced during the development of normal tissues, this finding may represent a regenerative response of cartilage after OA-inducing damage. In this context, it is of interest that the production of syncytin 1, which is the *env* gene product of HERV-W, was confirmed in these OA chondrocytes. Syncytin 1 is primarily a viral envelope glycoprotein that plays a role in infection, but it is expressed in the placenta and involved in trophoblast fusion. This protein is able to induce cell fusion; hence, its pathogenic activity may be suspected. Activation of HERV in OA chondrocyte was further supported by the viral particle formation confirmed by electron microscopy, even though its pathological role is questionable. Of note, recent studies suggest the involvement of lncRNAs, including the HERV-derived UCA1, in OA pathogenesis [90,91].

### 7.2. Rheumatoid Arthritis

Rheumatoid arthritis (RA) is another major disorder in synovial joints, with progressive destruction of joint tissues, including articular cartilage. In this systemic inflammatory disease with an autoimmune background, cartilage is degenerated as a result of inflammatory responses, in which synovial cells play a major role [92]. The etiological involvement of human retroviruses in RA was initially suspected, since transgenic mice with human T cell lymphotropic virus type I (HTLV-I) were shown to develop RA-like arthropathy [93]. In relation to this notion, retrotransposons attracted the interest of the researchers in this field as possible etiological factors for RA. With regard to the LTR type, a molecular genetic study found that a HERV-K LTR insertion into the HLA-DQB1 locus was associated with RA development [94]. Several other studies reported the traces of retroviral transcripts and particles in synovial fluids and tissues from RA patients [95]. Concerning the non-LTR type, it was once described that ORF1 protein and ORF2 transcripts from LINE-1 retrotransposons were detected in the synovial tissues of five out of six RA patients, whereas three OA and two normal synovial tissues showed no signals [96]. Overexpression of LINE-1 in RA synoviocytes caused the induction of several genes, including stress-induced protein kinase 2δ(SAPK2δ), which might provoke catabolic action of synoviocytes to harm articular cartilage [96]. However, mechanistic involvement of these LTR-type and non-LTR-type retrotransposons in RA pathogenesis has not been established thus far.

## 8. Conclusive Remarks

It is now obvious that retrotransposons have made a significant contribution to the generation of mammalian species by modifying and enlarging the genome. Particularly, considering their extraordinary genome occupancy, no humans with present shape, which is determined mostly by endochondral ossification, were present without HERV, LINEs, and SINEs. In addition, the fact that the genomes of crustacean and insects contain a number of retrotransposons suggests their contribution to intramembranous ossification—the older form of bone formation [97,98]. Thus, retrotransposons may be manipulating our craniofacial development by directly regulating osteoblasts as well. These mobile genetic elements are still actively editing our genome, indicating that these elements may still forward mammalian species toward further evolution.

Our cells, particularly germ cells, are armed by a protection system against the expansion of retrotransposons in the genome. Typically, using fossils of retrotransposons, small RNAs are produced that specifically recognize them, which lead to the silencing of relevant elements. These small RNAs consist of endogenous small interfering RNA (esiRNA) [99], miRNA, and PIWI-interacting RNA (piRNA) [100]. Among these RNAs, piRNAs are thought to be the most important in protecting our genome from inheritable assault by retrotransposons, as they are specifically produced in germ and embryonic cells. Despite such protection systems, mammalian genomes are still being edited by these endogenous parasites, as exemplified in this review.

In addition, we should stay alert to exogenous retroviruses as well, especially to human immunodeficiency viruses (HIVs). Considering the property of HIVs as lentiviruses, their genome-editing activity is evident. Although their primary targets are T-lymphocytes and macrophages, possible infection to other types of cells may not be ruled out. Indeed, direct interaction with sperm cells and HIV-1 virions via mannose receptor was observed [101], suggesting possible invasion of HIV into the genome of human embryo. In spite of the establishment of anti-retroviral therapy (ART) against AIDS, one should be careful to avoid viral contact itself, since our shape in the future with HIV in our genome is unpredictable.

## Abbreviations

ERV	Endogenous retrovirus
LINE	Long interspersed nuclear element
SINE	Short interspersed nuclear element
